# Endophenotypical drift in Huntington’s disease: a 5-year follow-up study

**DOI:** 10.1186/s13023-021-01967-2

**Published:** 2021-08-03

**Authors:** Marie N. N. Hellem, Rebecca K. Hendel, Tua Vinther-Jensen, Ida U. Larsen, Troels T. Nielsen, Lena E. Hjermind, Esben Budtz-Jørgensen, Asmus Vogel, Jørgen E. Nielsen

**Affiliations:** 1 The Neurogenetics Clinic, Danish Dementia Research Centre, Rigshospitalet, University of Copenhagen, Blegdamsvej 9, 2100 Copenhagen, Denmark; 2 Department of Psychology, University of Copenhagen, Øster Farimagsgade 2, 1014 Copenhagen, Denmark; 3 Department of Neurology, Bispebjerg-Frederiksberg Hospital, Bispebjerg Bakke 23, 2400 Copenhagen NV, Denmark; 4 Department of Public Health, Section of Biostatistics, University of Copenhagen, Øster Farimagsgade 5, 1014 Copenhagen, Denmark

**Keywords:** Huntington’s disease, Endophenotype, Psychiatric symptoms, Cognitive symptoms

## Abstract

**Background:**

Huntington’s disease (HD) is clinically characterized by progressing motor, cognitive and psychiatric symptoms presenting as varying phenotypes within these three major symptom domains. The disease is caused by an expanded CAG repeat tract in the huntingtin gene and the pathomechanism leading to these endophenotypes is assumed to be neurodegenerative. In 2012/2013 we recruited 107 HD gene expansion carriers (HDGECs) and examined the frequency of the three cardinal symptoms and in 2017/2018 we followed up 74 HDGECs from the same cohort to describe the symptom trajectories and individual drift between the endophenotypes as well as potential predictors of progression and remission.

**Results:**

We found higher age to reduce the probability of improving on psychiatric symptoms; increasing disease burden score ((CAG-35.5) * age) to increase the risk of developing cognitive impairment; increasing disease burden score and shorter education to increase the risk of motor onset while lower disease burden score and higher Mini Mental State Examination increased the probability of remaining asymptomatic. We found 23.5% (N = 8) to improve from their psychiatric symptoms.

**Conclusions:**

There is no clear pattern in the development of or drift between endophenotypes. In contrast to motor and cognitive symptoms we find that psychiatric symptoms may resolve and thereby not entirely be caused by neurodegeneration. The probability of improving from psychiatric symptoms is higher in younger age and advocates for a potential importance of early treatment.

**Supplementary Information:**

The online version contains supplementary material available at 10.1186/s13023-021-01967-2.

## Background

Huntington’s disease (HD) is an autosomal dominant, progressing neurodegenerative disease. Clinically, the disease is characterized by a triad of symptoms: motor disturbances, neuropsychiatric manifestations and cognitive impairment. The clinical HD diagnosis relies on unambiguous extrapyramidal motor symptoms that cannot be explained by any other movement disorder even though both cognitive and psychiatric symptoms often precede this by years [1]. The motor symptoms in HD typically debut in the fourth or fifth decade of life and gradually worsen over 10–20 years until death. The presence of non-motor symptoms has gradually been more accepted as early symptoms of HD and become targets of disease modulating treatments; however, the pathways leading to the different clinical endophenotypes within HD are still not fully understood. Several findings suggest that both neuropathology, environmental stress and genetic variants other than the causative CAG repeat expansion in the Huntingtin gene contribute to the occurrence of cognitive impairment and neuropsychiatric symptoms [2–4]. The understanding of the development and assessment of these non-motor symptoms needs to be exact to correctly ascertain the degree and progression of both cognitive and psychiatric symptoms. Several longitudinal studies have been carried out with focus on symptom development in premanifest Huntington’s disease gene expansion carriers (HDGECs); however, these studies have described the different symptom trajectories on a group level.

The classical presentation of motor symptoms consists of involuntary movements, chorea, which begins early in the disease course and gradually the hyperkinetic movements become more slow and akinesia and rigidity evolve [1], and this motor dysfunction strongly correlates with the volume of the striatum and white matter [5]. The typical cognitive deficits are mental slowing, decreased attention, flexibility, planning, visuospatial functions and emotion recognition [5]. Cognitive impairment is thought to be caused by dysfunction of the frontostriatal circuits due to gradual degeneration of the caudate nucleus [6], however, studies on the evolvement of cognitive impairment in HD are inconclusive. In the TRACK-HD study, the impairment was measured years before motor onset and significant progression of the decline across 36 months in the premanifest HDGEC was reported [7] while other studies have found the rate of cognitive change in HDGECs to be similar to that in non-carriers [8].

Psychiatric symptoms including depression, irritability, apathy, perseveration, obsession/compulsion and psychosis are described as more variable than the cognitive and motor symptoms and little or no correlation has been described between structural imaging and psychiatric symptoms [5]. Cross sectional studies have shown HDGECs to have a lifetime prevalence between 33% and 76% of one or more of these symptoms [4]. One longitudinal study even showed that 99% of 111 HDGECs had neuropsychiatric symptoms occurring during a 3 year follow-up period [9]. While psychiatric symptoms have been found to worsen over time, the most striking single psychiatric feature demonstrating clear longitudinal progression, significantly associated with cognitive and functional decline, is apathy [7], and subtle psychiatric symptoms have been described more than 10 years before expected motor onset [10]. The psychiatric symptoms were reported to progress with disease severity and thereby likely to be part of the neurodegenerative disease process [11], but no longitudinal studies have described the progression or remission of symptoms on the individual level.

We have previously proposed a clinical classification that embraces the HDGECs with non-motor-symptoms [12] and here we describe the individual endophenotypical drift of symptoms during an average of 5.5 and a minimum of 4.5 years in a HDGEC follow-up cohort, and potential predictors of progression and remission.

## Materials and methods

From January 2012 to March 2013 we recruited 107 HDGECs (Cohort 2013) from the Neurogenetics Clinic, Danish Dementia Research Centre, Rigshospitalet, Copenhagen, Denmark. All were examined by a physician and a neuropsychologist and evaluated regarding motor symptoms, neuropsychiatric symptoms, and cognitive impairment. All individuals were confirmed HDGECs and had a Unified Huntington’s Disease Rating Scale-99, (UHDRS-motor) total motor score ≤ 55, a Mini Mental State Examination (MMSE) score ≥ 24, and a Montreal Cognitive Assessment (MoCA) score ≥ 19 [13].

From June 2017 to January 2019 we followed-up as many as possible from Cohort 2013. The group of participants who was part of Cohort 2013 and participated again in the present follow-up study is referred to as either Cohort 2018 when their follow-up values are described or Baseline 2013 cohort when their data from 2013 is described.

We were able to follow-up 74 participants and characterized the endophenotypical drift of these participants.

The study was approved by the Ethics committee of the Capital region of Denmark (H-17002606) and written informed consent was obtained from each participant before enrollment.

### Clinical evaluation

The UHDRS was used for evaluation of motor signs in both cohorts [14]. A UHDRS-motor score > 5 classified as motor manifest and a UHDRS-motor score of 5 or less as premanifest HDGEC. The participants were assessed with UHDRS Total Functional Capacity (TFC), UHDRS Function scales and short Problem Behaviors Assessment (PBA-s). Previous and present medical and psychiatric history was recorded.

### Neuropsychological testing and classification

To exclude participants who were not eligible for the neuropsychological tests, all participants were screened with MMSE and MoCA. Participants who, as part of the 2018 cohort participation, scored ≤ 24 on MMSE and ≤ 19 on MoCA were classified as cognitively impaired and not candidates for neuropsychological testing.

In the neuropsychological assessment memory, psychomotor speed/attention, executive functions and visuospatial functions were examined. The applied tests are listed in Table 1. The normative data for these tests was extracted from a database at the Department of Neurology, Rigshospitalet, University of Copenhagen from 80 age-matched healthy subjects. For further details see Vinther-Jensen et al. [13] and Table 1.

**Table 1:** Classification and evaluation of participants

Clinical evaluation	UHDRS total motor score (TMS)	
	Pre-manifest: TMS ≤ 5	Manifest: TMS > 5
**Neuropsychiatric evaluation** *Allocation to neuropsychiatric group if at least one criterion is met*	1. Usage of psychotropic medication on neuropsychiatric indication	
	2. A SCL-90-R GSI T-score ≥ 63 or a T-score ≥ 63 in more than two of the nine primary symptom dimensions. The SCL-90-R cut-offs are based on SCL-90-R guide	
	3. A HAM-17 score ≥ 13 (moderate to severe depression)	
**Cognitive evaluation** *Cognitively impaired if*	*Pre-morbid intellectual level:*	Wechsler Adult Intelligence Scale (WAIS)
		Danish adult reading test (DART)
*A. four or more test performances were impaired*	*Memory:*	Selective reminding test
		Rey complex figure text
*B. all tests in a domain (except psychomotor speed/attention) were impaired*	*Psychomotor speed/attention:*	Trail making test A & B
		SDMT
	*Executive functions:*	Stroop test (100 items − incongruent))
		Verbal fluency tests (category fluency (animals, 1 min), lexical fluency (s-words and a-words, 1 min))
*C. performances on all tests in the psychomotor speed/attention domain, and at least one other test was below cut-off*	*Visiospatial functions:*	Rey complex figure
		Ravens progressive matrices (set 1)
		Block design test (modified version)

Classification of participants into groups of premanifest and manifest HD gene expansion carriers and evaluation of neuropsychiatric symptoms and cognitive impairment

*SCL-90-R* Symptom Checklist-90-Revised, *HAM-17* Hamilton Rating Scale for Depression-17, *SDMT* Symbol digit modality test

### Neuropsychiatric assessment and classification

Neuropsychiatric symptoms were assessed by the Symptom Checklist-90-Revised (SCL-90-R) and the Hamilton Rating Scale for Depression-17 (HAM-17). The SCL-90-R is a questionnaire with 90 items reflecting current psychological symptoms rated by the Likert scale of distress, ranging from “not at all” to “extremely”. The 90 items are divided into nine primary symptom dimensions: somatization (SOM), obsessive–compulsive (O-C), interpersonal sensitivity (I-S), depression (DEP), anxiety (ANX), hostility (HOS), paranoid anxiety (PHOB), paranoid ideation (PAR) and psychoticism (PSY). The SCL-90-R also provide three global indices of distress: Global Severity Index (GSI), Positive Symptoms Distress Index (PSDI), and positive symptoms Total (PST). The scores from the questionnaire can be converted to T-scores normalized to a Danish sample of non-psychiatric individuals sorted by gender; higher T-scores indicating more psychiatric distress.

The HAM-17 was applied as a semi-structured interview covering 17 symptom areas and participants were allocated to the neuropsychiatric group as indicated in Table 1.

### Data analysis

Comparing the premanifest and manifest HDGECs, we analyzed the demographic data, age, gender, CAG repeat, Disease Burden score ((CAG–﻿35.5) * age) [15] and results from SDMT and MoCA and MMSE using Mann–Whitney U test for the continuous data and Chi square test or Fischer’s exact test for the categorial data.

We compared the scores obtained in the neuropsychiatric and neuropsychological tests between the premanifest and manifest HDGECs of Cohort 2018. The comparisons were based on t-test.

We investigated the endophenotypical groups for predictors of potential drift towards progression, regression or stability in symptoms. We examined all observed endophenotypical drifts for effects of age, gender, CAG repeat number, Disease Burden score, education and, if relevant, presence of psychiatric, cognitive and motor symptoms. We used logistic regression analysis and effects are presented as Odds Ratios (OR). The analyses were conducted in SAS Enterprise Guide version 7.1 and *p* values of 0.05 or less were considered significant.

## Results

Cohort 2013 included 107 HDGECs and Cohort 2018 74 HDGECs (Table 2). Six HDGECs from Cohort 2013 had died and 27 did not participate in Cohort 2018. We were not able to get into contact with 7 Cohort 2013 participants and the remaining 20 either felt too ill, did not have the time or did not respond after receiving information about the project.

**Table 2:** Demographic data

	Premanifest HD gene-expansion carriers	Motor manifest HD gene-expansion carriers	Level of significance *p* values	Premanifest HD gene-expansion carriers	Motor manifest HD gene-expansion carriers	Level of significance *p* values
	Cohort 2013			Cohort 2018		
Number	51	56		30	44	
Gender m/f	30/21	33/23	0.32	17/13	25/19	1.0
Age at examination (years)	36 (20–54)	50 (24–75)	< 0.001	40.5 (30–60)	53.5 (29–80)	0.0002
CAG repeat length	42 (39–48)	43 (40–53)	< 0.38	41 (39–48)	43 (40–53)	0.0052
Disease Burden scores	234.0 (108.0–437.0)			241.3 (133.0–437.5)		
UHDRS-motor (score)	2 (0–5)	21 (6–51)	< 0.001	1 (0–5)	32 (7–91)	< 0.0001
TFC (score)	13 (11–13)	10 (4–13)	< 0.001	13 (11–13)	8 (0–13)	< 0.0001
MMSE (score)	29 (26–30)	28 (24–30)	< 0.001	29.5 (27–30)	28 (20–30)	< 0.0001
MoCA (score)	28 (25–30)	25 (19–30)	< 0.001	30 (23–30)	25 (12–30)	< 0.0001

All data is presented as median (range)

Disease Burden score ((CAG–﻿35.5) * age)

*HD* Huntington’s disease, *m/f* male/female, *TFC* total function capacity, *MMSE* Mini Mental state examination, *MoCA* Montreal Cognitive Assessment

In Cohort 2018, 30 of the participants were premanifest and 44 were motor manifest. The motor manifest HDGECs were significantly older than the premanifest HDGECs, had longer CAG repeats, lower scores on the TFC, the MMSE and MoCA.

### Cohort 2018

#### Cognitive symptoms

In Cohort 2018, 72.7% of the motor manifest and 10% of the premanifest HDGECs were cognitively impaired according to our criteria.

#### Neuropsychiatric symptoms

In Cohort 2018, 16.7% of the premanifest HDGECs and 63.6% of the manifest HDGECs were classified as having psychiatric symptoms.

**Fig. 1:**
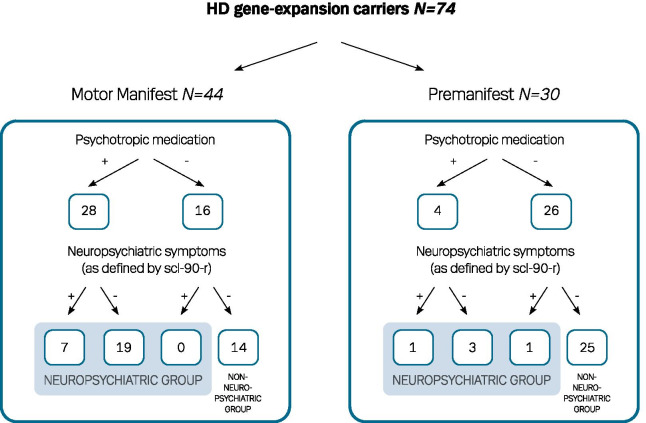
Flowchart used for categorization of participants into the neuropsychiatric vs. the non-neuropsychiatric groups. Psychotropic—meaning use of psychotropic medication in the motor manifest group, 4 participants did not complete the SCL-90-R, 2 received psychotropic medication and therefore classified as having psychiatric symptoms and 2 did not receive psychotropic medication and were clinically evaluated as not having psychiatric symptoms

We found 13.3% of the 30 premanifest HDGECs (4 participants) to be treated with psychotropic medication and one of these had neuropsychiatric symptoms according to the SCL-90-R (Fig. 1). Among the 86.7% who did not use psychotropic medication one had neuropsychiatric symptoms according to the SCL-90-R. In the motor manifest group (Fig. 1), 36.4% did not use psychotropic medication and none of these HDGECs had neuropsychiatric symptoms according to SCL-90-R. 63.6% were treated with psychotropic medication and 25% of these had neuropsychiatric symptoms according to SCL-90-R.

Comparing the neuropsychiatric symptoms of the motor manifest and the premanifest on the SCL-90-R and HAM-17 we found 7 out of 13 parameters to be significantly higher in the motor manifest group. After Bonferroni correction we only found a significant difference on the symptom dimensions O-C and DEP (Table 3).

**Table 3:** Neuropsychiatric symptoms and neuropsychological test performance in the premanifest and the motor manifest gene-expansion carriers

	Premanifest HD gene-expansion carriers	Motor manifest HD gene-expansion carriers	Level of significance *p* values
	N = 30	N = 44	
*SCL-90-R*			
Global severity Index	42.5 (9.7)	49.4 (11.0)	0.0083*
Positive symptoms total	42.8 (9.2)	48.3 (10.2)	0.0232*
Positive Symptoms Distress Index	44.1 (12,6)	52.4 (14.8)	0.0165*
Somatization	46.2 (9.2)	48.9 (9.9)	0.2385
Obsessive–compulsive	42.3 (10.3)	51.1 (10.6)	0.0009*
Interpersonal sensitivity	43.0 (9.9)	46.9 (10.7)	0.1218
Depression	42.7 (9.1)	50.3 (10.6)	0.0024*
Anxiety	43.7 (9.3)	49.2 (10.3)	0.0252*
Anger-hostility	46.9 (10.3)	49.2 (11.8)	0.3918
Phobic anxiety	45.6 (6.4)	51.6 (11.5)	0.0075*
Paranoid ideation	43.7 (8.3)	46.7 (10.1)	0.1945
Psychoticism	44.9 (7.5)	49.2 (11.1)	0.0525
Hamilton-17	2.0 (1.8)	3.2 (3.5)	0.07

	N = 30	N = 41	
Trail a (s)	20.0 (6.7)	53.4 (31.5)	< 0.0001*
Trail b (s)	45.7 (15.5)	140.6 (102.3)	< 0.0001*
SDMT (number of correct)	53.1 (10.3)	29.9 (14.5)	< 0.0001*
Stroop interference (sec)	109.3 (24.9)	181.5 (96.4)	0.0003*
Category verbal fluency	24.2 (4.3)	16.6 (6.8)	< 0.0001*
Lexical fluency, s-words	17.4 (4.7)	10.2 (6.2)	< 0.0001*
Lexical fluency, a-words	11.3 (4.2)	7.4 (3.9)	0.0004*
Reys complex figure test, recall	24.6 (7.1)	18.1 (5.8)	0.0003*
Reys complex figure test, copy	35.3 (1.1)	32.4 (4.5)	0.001*
Block design (number correct)	11.9 (0.4)	11.1 (1.9)	0.03*
Raven’s advanced progressive matrices	10.0 (2.0)	6.7 (2.1)	< 0.0001*
Selective reminding test, immediate recall (no of errors)	8.4 (5.5)	31.1 (18.9)	< 0.0001*
Selective reminding test, delayed recall	8.4 (1.1)	6.1 (2.2)	< 0.0001*

*HD* Huntington’s disease, *SCL-90-R* Symptom Checklist-90-Revised, *SDMT* Symbol Digit Modality test; Data shown as median (SD); *p* values were calculated by independent sample t-test; After Bonferroni correction only obsessive–compulsive and depression remained significant

#### Endophenotypes

**Fig. 2:**
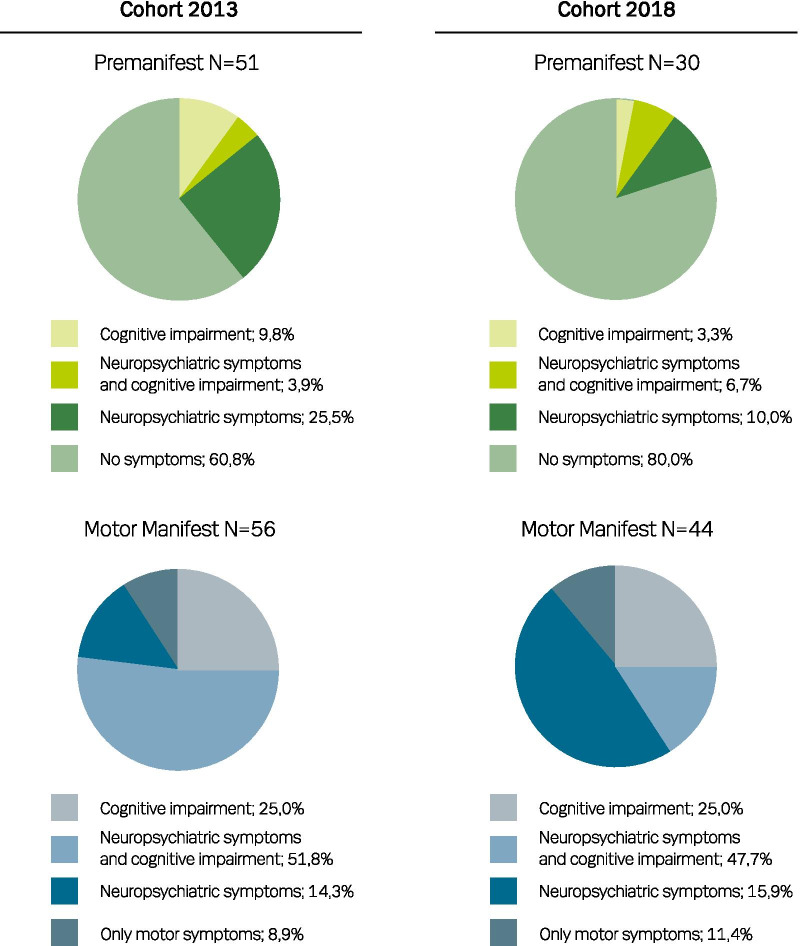
Distribution of the endophenotypes in Cohort 2013 and Cohort 2018

The distribution of the endophenotypes in the two cohorts is illustrated in Fig. 2.

The premanifest HDGEC endophenotype distribution in the two cohorts differ; in both groups the participants with no symptoms (Pre-0) form the largest group and the group classified with psychiatric symptoms (Pre-P), the second largest. In Cohort 2018, the participants classified with both psychiatric and cognitive symptoms (Pre-PC) form the third largest group which was the smallest group in Cohort 2013, and hence, the group classified with cognitive impairment (Pre-C) is the smallest in Cohort 2018.

The endophenotype distribution of the motor manifest participants were very alike in Cohort 2013 and Cohort 2018. Approximately half in both cohorts had a combination of both neuropsychiatric and cognitive symptoms (Man-PC). The second largest group are the participants classified with cognitive impairments (Man-C) followed by the group of HDGECs classified with neuropsychiatric symptoms (Man-P) and HDGECs with only motor symptoms (Man-0).

In the motor manifest group, we found older age, male gender, higher CAG repeat number and Disease Burden score to increase the risk of being classified as having both psychiatric symptoms and cognitive impairment, although not statistically significantly. There was a significant risk of being classified with both neuropsychiatric symptoms and cognitive impairment with higher UHDRS motor score (OR = 1.041; *p* = 0.0175) and lower scores for the following, TFC (OR = 0.756; *p* = 0.0103), MMSE (OR = 0.676; *p* = 0.0175) and MoCA (OR = 0.786; *p* = 0–0126). In the premanifest group female gender, higher CAG repeat number, higher Disease Burden score, higher UHDRS motor score and lower TFC, MMSE and MoCA increased the risk of being classified as having cognitive impairment, neuropsychiatric symptoms or both; However, only the lower TFC (OR = 0.060; *p* = 0.0284) was significant.

### Endophenotypical drift

**Fig. 3:**
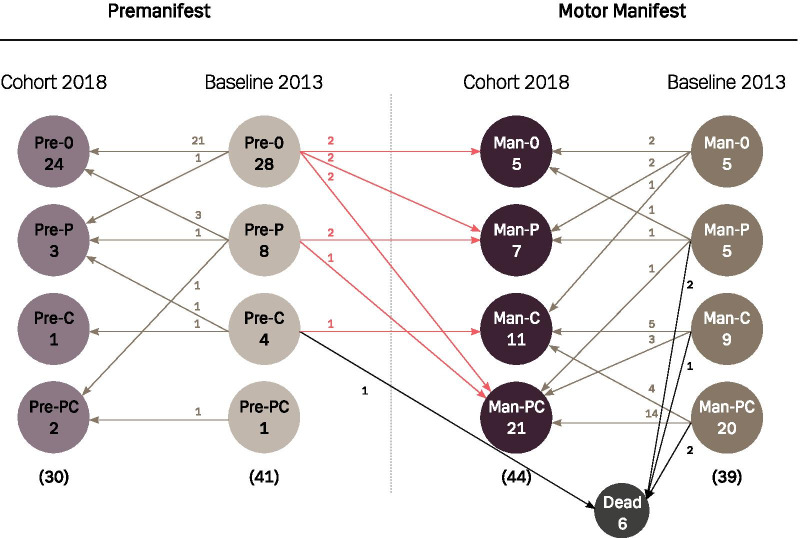
Endophenotypical drift in the Baseline 2013 Cohort to Cohort 2018. Overview of the follow-up participants and their drift between phenotypes. Right side of the figure represents the Manifest group (subgroups marked by a Man) and the left side the Premanifest group (subgroups marked by a Pre). Both sides are vertically divided into Baseline 2013 and Cohort 2018. The cohorts are horizontally divided in to four subgroups. The zero indicates neither cognitive nor psychiatric symptoms, P means addition of psychiatric symptoms, C addition of cognitive symptoms and PC addition of both cognitive and psychiatric symptoms. The number indicates the HDGECs in the group and the arrows show the drift from Baseline 2013

As illustrated in Fig. 3 there is no clear pattern in the individual endophenotypical drift between the two cohorts; 21 premanifest HDGECs remain asymptomatic, 10 premanifest HDGECs become motor manifest, 11 HDGECs are classified as having psychiatric symptoms and 8 are no longer classified as having psychiatric symptoms. Six HDGECs become classified as having cognitive impairment, one participant moves from being classified as cognitively impaired to being regarded as cognitively intact and 6 HDGECs die.

#### Psychiatric symptoms

The proportion of participants classified as having psychiatric symptoms in Cohort 2018, Pre-P, Pre-PC, Man-P and Man-PC, see Fig. 3 (44.6%), equals the proportion in the Baseline 2013 cohort (42.5%). Twenty-two percent of the premanifest Baseline 2013 cohort participants and 64% of the manifest Baseline 2013 cohort participants have been classified as having psychiatric symptoms.

Eleven (23.5%) HDGECs changed from having no psychiatric symptoms to being classified with psychiatric symptoms during the mean of 5.5 years. We found no significant risk factors for predicting this symptom development. The 8 (23.5%) HDGECs who recovered from being classified as having psychiatric symptoms show that the higher the age, the lower probability for improving on the classification of having psychiatric symptoms (OR = 0.892; *p* = 0.0303). Of the 8 who recovered from being classified as having psychiatric symptoms, 1 had a positive Hamilton-17, 7 had SCL-90-R registered symptoms, 6 were using psychotropic medication on neuropsychiatric indication. Five were motor manifest and 3 premanifest and 4 were classified as having cognitive impairment. Their mean age was 35.8 years, mean CAG repeat number 43 and Disease Burden score 321.2.

#### Cognitive impairment

Among the participants who participated in both Cohort 2013 and Cohort 2018, the proportion of participants classified as being cognitively impaired was in 2013 12.2% of the premanifest HDGECs and 74.4% of the manifest HDGECs. In Cohort 2018 we found 10% of the premanifest and 72.7% of the manifest HDGECs to be classified as having cognitive impairments.

Increasing Disease Burden score (OR = 1.019; *p* = 0.0132) was a significant risk factor for a change in classification from normal cognition to cognitive impairment.

#### Motor symptoms

Fifty-two percent of Cohort 2013 was motor manifest and 59.5% of Cohort 2018 was motor manifest. Twenty-four percent (N = 10) of the premanifest HDGECs who participated in both Cohort 2013 and Cohort 2018 changed status from premanifest to motor manifest during the 5.5 years (see Fig. 3).

We found a significantly increased risk of becoming motor manifest with increasing Disease Burden score (OR = 1.014; *p* = 0.0112) and shorter education (OR = 3.968; *p* = 0.0060).

#### HDGECs remaining asymptomatic

Fifty-one-point two percent (N = 21) of the premanifest HDGECs were still asymptomatic after 5.5 years. When comparing these 21 HDGECs to the 7 asymptomatic HDGECs who during the follow-up period were classified with psychiatric, cognitive or motor symptoms, we find them to have significantly shorter CAG repeat numbers (*p* = 0.0295), lower Disease Burden score (*p* = 0.0086) and higher MMSE score (*p* = 0.0007). Comparing them to all the premanifest HDGECs we found a significant increased probability of remaining asymptomatic with lower Disease Burden score (OR = 0.985; *p* = 0.0209), higher SDMT score (OR = 1.203; *p* = 0.0358) and longer education (OR = 2.422; *p* = 0.0211).

#### The deceased HDGECs

Of Cohort 2013, 5.6% have died. Five motor manifest and one premanifest, who did not differ from the motor manifest HDGECs neither in age, UHDRS or Disease Burden score (data not shown). We do not know the exact cause of death, but unsurprisingly we find the risk of dying to increase significantly with the increase of age (OR = 1.089; *p* = 0.0181) and Disease Burden score (OR = 1.009; *p* = 0.0193).

### Participants lost to follow-up

Twenty-seven participants were not part of Cohort 2018, 10 premanifest and 17 manifest HDGECs. They did not differ significantly demographically from the remaining participants from Cohort 2013 (see Additional file 1). The 10 premanifest HDGECs, who did not participate in the follow-up study, represented 38.5% of the Pre-P group from Cohort 2013, 20% of the Pre-C group, 50% of the Pre-PC and 9.6% of the Pre-0 group. The 17 manifest HDGECs represent 37.5% of the Man-P in Cohort 2013, 35.7% of Man-C and 27.6% of the Man-PC.

## Discussion

We describe a single site follow-up study of HDGECs over an average period of 5.5 years and emphasize the individual variability in the drift between clinical endophenotypes of HD. The clinical endophenotypes are based on a comprehensive assessment battery of the participants and this along with the relatively large patient group makes this study unique. We confirm an increased risk of motor onset and development of cognitive impairments with increasing Disease Burden [16]. Increasing neurodegeneration has been reported with increasing Disease Burden [5] and a correlation between cognitive symptoms and white matter atrophy has also been shown [17] which is in line with our findings of increasing Disease Burden with increased risk of onset of motor and cognitive symptoms. Furthermore, our study highlights the unpredictability of the psychiatric symptoms, however, we are not able to point to a causality of these symptoms. We observe that the psychiatric symptoms may evolve and resolve; the only significant predictor being increasing age which reduces the probability of improving from the psychiatric symptoms. The decreasing potential of psychiatric improvement with increasing age may, however, reflect the progressive neurodegeneration and presumed decreased brain plasticity in HD [18].

Neurodegenerative diseases like Parkinson’s disease and Alzheimer’s disease also encompass more than one clinical endophenotype. In Parkinson’s disease there have been identified different genetic variants for different endophenotypes with a different treatment response and prognosis [19]. We found the phenotypes in HD to change over time, not only like the more recent understanding of the disease progression in HD with psychiatric symptoms and cognitive impairment starting years before onset of motor symptoms and all three in constant aggravation. We found, however, the phenotype more versatile; some participants debut with pure motor symptoms while others debut with the full triad of symptoms and the psychiatric symptoms are very variable during the disease course. This makes it unlikely that the endophenotypes are explained by the CAG repeat expansion variant alone. Genetic polymorphisms affect age of onset as well as cognitive impairment and psychiatric symptoms [3], but also environmental factors, like physical activity and alcohol consumption, have been found to modulate onset and progression of HD [20] and pharmacological treatment can affect the different symptoms as well [21].

In Alzheimer’s disease the symptoms progress within a spectrum of cognitive impairment that expands from normal to end-stage dementia, which has been linked to the biological spread of the disease [22]. In HD we also see a spread of pathology with the earliest changes seen in the Caudate nucleus and the Putamen as loss of medium spiny neurons, followed by white matter loss around the striatum, corpus callosum and posterior white matter tracts and then a more pronounced and widespread grey matter loss [7, 23–25]. While the motor symptoms are correlated to the changes in the striatum, the mood and behavioral symptoms of HD have been suggested to reflect the changes in the limbic circuit [26] and dysfunction in the basal-ganglia-thalamocortical loops [27].

Describing the trajectories of the major symptom domains in our study we, as expected, find the number of motor manifest participants increased during the 5.5 years. The association, that we confirm, between motor symptoms and Disease Burden score is well established, but environmental factors has also been described to affect age of onset [28] which could be in line with our finding of the risk of motor symptom onset to increase with lower education. But whether it is in fact higher education that protects against progression by increasing the plasticity of the brain [29] or the correlation reflects that a longer CAG repeat facilitates a lower education is not known.

The proportion of participants classified as being cognitively impaired also increased during the follow-up period, 40.5% in the Baseline 2013 cohort and 47.3% in Cohort 2018. The increase in frequency of participants classified as having cognitive impairment is in accordance with previous studies [5]. However, one premanifest HDGEC drifted from being classified as cognitively impaired to not being cognitively impaired. This participant developed psychiatric symptoms during the 5.5 years of follow-up. Clinical records show, that this participant in 2013 was affected by external stress factors at the time of testing, which has likely affected the performance on cognitive tests. Our classification is based on a thorough examination that evaluates the individual participant, but there will still be some participants, estimated 5%, with incorrect cognitive classification.

The frequency of participants classified as having psychiatric symptoms in Cohort 2018 (44.6%) equals the distribution among the Baseline 2013 cohort (42.5%), despite the expected progression in symptoms over time. While 11 HDGECs drifted to the group being classified as having psychiatric symptoms, we found that 8 HDGECs recovered from being classified as having psychiatric symptoms. The improvement in psychiatric symptoms can be a result of pharmacologic treatment but after ended treatment the symptoms are still absent which is surprising in the context of psychiatric symptoms supposed to be caused by neurodegeneration [30]. A longitudinal study investigating psychiatric symptoms in prodromal HD found psychiatric symptoms to increase with progression of the disease based on companion reports showing worsening of symptoms in the HDGEC, while the lack of reported worsening of symptoms could be explained by decreasing awareness in the HDGEC [11]. Another study found apathy to correlate with the underlying disease process, while depression only tended to correlate with the proximity to the clinical onset [31]. In our cohorts, the participants who improved on their psychiatric status are from both the premanifest and manifest group, none of them were apathetic clinically nor according to the PBA-s. Using their mean CAP scores [32] we estimated them to be approximately 6 years from expected motor onset, none of the premanifest participants being cognitively affected or having lost disease awareness.

As a consequence of this being a follow-up study, we expected our HDGECs in Cohort 2018 to be older than in Cohort 2013; in both cohorts the premanifest HDGECs were significantly younger and had significantly lower UHDRS motor scores and higher TFC, MMSE and MoCA scores. In Cohort 2018, the premanifest HDGECs had significantly shorter CAG repeats than the manifest HDGECs, which is in accordance with the premanifest HDGECs who become motor manifest have significantly higher Disease Burden score (*p* = 0.0047) and significantly longer CAG repeats (*p* = 0.022) than the premanifest HDGECs who remain premanifest. Twenty-five percent of Cohort 2013 were not able to participate in this follow-up study; however, they did not differ significantly in demographics compared to the Baseline 2013 cohort. Cohort 2013 consisted of premanifest to moderately affected HDGECs and Cohort 2018 will be somewhat predefined, and therefore our results cannot be compared to cross sectional studies. This limitation does, however, not inflict on the unpredictability of HD or the importance of a thorough examination of the HDGECs to clarify their endophenotype to optimize treatment and support.

## Conclusion

We describe the individual unpredictability in the progression of symptoms in HD as seen in Fig. 3. Therefore, we recommend regular follow-up where symptoms can be identified and potentially relieved.

More than one fifth of the HDGECs who were classified as having psychiatric symptoms in the Baseline 2013 cohort improved from their symptoms. Six of these participants were using psychotropic medication at baseline and none at follow-up. If the symptoms were only to be caused by neurodegeneration, we would expect them to last and worsen after ended pharmacological treatment. However, other studies suggest that the psychiatric symptoms often disappear as the disease progresses because of increasing lack of disease awareness [11], which does not seem to be the case in our study. We found the probability of improving from psychiatric symptoms to be significantly higher with younger age suggesting a potential importance of early treatment of psychiatric symptoms in younger individuals.

## Supplementary Information

**Additional file 1.** Demographic data on the lost to follow-up participants.

## Data Availability

The datasets used and analyzed during the current study are available from the corresponding author on reasonable request.
