# Supplementary material for: Endophenotypical drift in Huntington’s disease: a 5-year follow-up study
Source: Orphanet J Rare Dis. 2021 Aug 3;16:340. doi: 10.1186/s13023-021-01967-2 (PMC8336065; doi:10.1186/s13023-021-01967-2)
Supplement: Supplementary file 1 — Additional file 1. Demographic data on the lost to follow-up participants. [file 13023_2021_1967_MOESM1_ESM.docx]

# SUPLEMENTARY

Table A

Demographic data on participants not able to participate in the follow-up cohort (data from 2013) and the participants who was part of the follow-up cohort (marked with grey), the participants who had died are not included.

|  | Premanifest HDGECs N=10 (19.6%) | Premanifest HDGECs N=40 | P-value  (Kruskal-Wallis) | Manifest HDGECs N=16 (28.6%) | Manifest HDGECs N=34 | P-value  (Kruskal-Wallis) |
| --- | --- | --- | --- | --- | --- | --- |
| Gender (m/f) | 7/3 | 22/19 |  | 9/7 | 20/14 |  |
| Age in 2013 | 36.5 (20-49) | 36 (24-54) | 0.72 | 52.5 (35-71) | 49.5 (24-75) | 0.49 |
| CAG repeat | 42.5 (40-47) | 41.5 (39-48) | 0.42 | 42 (40-48) | 43 (40-53) | 0.62 |
| Disease Burden score (Penney) | 243.5 (110-437) | 229.8 (108-378) | 0.56 |  |  |  |
| UHDRS motor score | 1.5 (0-4) | 2 (0-5) | 0.98 | 25 (11-39) | 17.5 (6-41) | 0.11 |
| TFC | 13 (11-13) | 13 (11-13) | 0.94 | 10 (7-13) | 10 (4-13) | 0.49 |
| MMSE | 29 (28-30) | 30 (26-30) | 0.09 | 28 (24-29) | 28 (25-30) | 0.20 |
| MoCA | 28.5 (26-30) | 28 (25-30) | 0.84 | 26 (24-30) | 25 (20-30) | 0.33 |
| No psychiatric or cognitive symptoms (N) | 3 (9.7%) | 28 |  | 0 | 5 |  |
| Psychiatric symptoms group (N) | 5 (38.5%) | 9 |  | 3 (37.5%) | 4 |  |
| Cognitive impairment group (N) | 1 (20%) | 4 |  | 5 (35.7%) | 9 |  |
| Cognitive and psychiatric group (N) | 1 (50%) | 1 |  | 8 (27.6%) | 21 |  |

Data presented as median and range

Number of HDGECs in the different classification groups are followed by their percentual share in their original group.
